# Prevalence of Mental Health Disorders Among Immigrant, Refugee, and Nonimmigrant Children and Youth in British Columbia, Canada

**DOI:** 10.1001/jamanetworkopen.2021.44934

**Published:** 2022-02-15

**Authors:** Anne M. Gadermann, Monique Gagné Petteni, Magdalena Janus, Joseph H. Puyat, Martin Guhn, Katholiki Georgiades

**Affiliations:** 1 Human Early Learning Partnership, School of Population and Public Health, The University of British Columbia, Vancouver, British Columbia, Canada; 2 Centre for Health Evaluation and Outcome Sciences, Providence Health Care Research Institute, Vancouver, British Columbia, Canada; 3 Offord Centre for Child Studies, Department of Psychiatry and Behavioural Neurosciences, McMaster University, Hamilton, Ontario, Canada; 4 School of Population and Public Health, The University of British Columbia, Vancouver, British Columbia, Canada

## Abstract

**Importance:**

There remains limited understanding of population-level patterns of mental disorder prevalence for first- and second-generation immigrant and refugee children and youth and how such patterns may vary across mental disorders.

**Objective:**

To examine the diagnostic prevalence of conduct, attention-deficit/hyperactivity disorder (ADHD), and mood/anxiety disorders in immigrant, refugee, and nonimmigrant children and youth in British Columbia, Canada.

**Design, Setting, and Participants:**

This retrospective, population-level cohort study examined linked health administrative records of children and youth in British Columbia (birth to age 19 years) spanning 2 decades (1996-2016). Physician billings, hospitalizations, and drug dispensations were linked to immigration records to estimate time-in–British Columbia–adjusted prevalence of mental disorder diagnosis among children and youth from immigrant or refugee backgrounds compared with those from nonimmigrant backgrounds. Analyses were conducted from August 2020 to November 2021.

**Main Outcomes and Measures:**

The diagnostic prevalence of conduct, ADHD, and mood/anxiety disorders were the main outcomes. Results were stratified by migration category (immigrant, refugee, nonimmigrant), generation status (first- and second-generation), age, and sex.

**Results:**

A total of 470 464 children and youth in British Columbia were included in the study (227 217 [48.3%] female). Nonimmigrant children and youth represented 65.5% of the total study population (307 902 individuals). Among those who migrated, 142 011 (87.8%) were first- or second-generation immigrants, and 19 686 (12.2%) were first- or second-generation refugees. Diagnostic prevalence of mental disorders varied by migration category, generation status, age, and sex. Children and youth from immigrant and refugee backgrounds (both first- and second-generation), compared with nonimmigrant youth, generally had a lower prevalence of conduct disorder (eg, age 6-12 years: first-generation immigrant, 2.72% [95% CI, 2.56%-2.90%] vs nonimmigrant, 7.03% [95% CI, 6.93%-7.13%]), ADHD (eg, age 6-12 years: first-generation immigrant, 4.30% [95% CI, 4.10%-4.51%] vs nonimmigrant, 9.20% [95% CI, 9.08%-9.31%]), and mood/anxiety disorders (eg, age 13-19 years: first-generation immigrant, 11.07% [95% CI, 10.80%-11.36%] vs nonimmigrant, 24.54% [95% CI, 24.34%-24.76%]). Among immigrant children and youth, second-generation children and youth generally showed higher prevalence of conduct, ADHD, and mood/anxiety disorders than first-generation children and youth (eg, ADHD among second-generation immigrants aged 6-12 years, 5.94% [95% CI, 5.75%-6.14%]; among first-generation immigrants aged 6-12 years, 4.30% [95% CI, 4.10%-4.51%]). Second-generation refugee children had the highest diagnostic prevalence estimates for mood/anxiety in the 3-to-5–year age range relative to first- and second-generation immigrant and nonimmigrant children (eg, second-generation refugee, 2.58% [95% CI, 2.27%-2.94%] vs second-generation immigrant, 1.78% [95% CI, 1.67%-1.89%]). Mental disorder diagnoses also varied by age and sex within immigrant, refugee, and nonimmigrant groups.

**Conclusions and Relevance:**

These findings show differences in diagnostic mental disorder prevalence among first- and second-generation immigrant and refugee children and youth relative to nonimmigrant children and youth. Further investigation is required into how cultural differences and barriers in accessing health services may be contributing to these differences.

## Key Points

**Question:**

What is the administrative data–derived diagnostic prevalence of mental disorders for immigrant, refugee, and nonimmigrant children and youth in British Columbia, Canada?

**Findings:**

In this cohort study that included 470 464 children and youth in British Columbia, Canada, children and youth from immigrant and refugee backgrounds (both first- and second-generation) had a significantly lower diagnostic prevalence of conduct disorder, attention-deficit/hyperactivity disorder, and mood/anxiety disorders than their nonimmigrant counterparts, with few exceptions.

**Meaning:**

In this study, the differences found in diagnostic mental disorder prevalence among first- and second-generation immigrant and refugee children and youth vs their nonimmigrant counterparts underline the importance of better understanding cultural differences as well as differential barriers in accessing health services.

## Teaser

This cohort study examines the diagnostic prevalence of conduct disorder, attention-deficit/hyperactivity disorder (ADHD), and mood/anxiety disorders in immigrant, refugee, and nonimmigrant children and youth in British Columbia, Canada.

## Introduction

For at least half of all mental health problems across the life course, onset is in childhood or adolescence.^1,2,3^ Understanding mental disorder prevalence prior to adulthood is critical for prevention and intervention,^2,3^ and without early treatment and support, difficulties are likely to persist over the long term, diminishing social, educational, and vocational prospects.^4^ Despite this knowledge, there remains a need for mental health interventions to take into account the contexts in which they are implemented.^5^ In the Canadian context, child and adolescent mental health strategies need to consider the large and growing immigrant and refugee population.^6^ As of 2016, 37.5% of children in Canada aged 14 years and younger were born outside of Canada or had at least 1 parent who was born outside of Canada (almost 2.2 million), a proportion projected to reach 50% by 2036.^7^

A contextualized understanding of the mental health of children and youth in Canada requires an understanding of mental disorder prevalence across immigrant, refugee, and nonimmigrant subpopulations. An epidemiological pattern known as the healthy immigrant effect (or the immigrant paradox) has emerged in Canada and other Western nations, whereby immigrant populations overall tend to show better health and mental health outcomes than nonimmigrant populations, but this tends to deteriorate over time spent in the country and across generations.^8,9^ The pattern and how it applies across subpopulations and settlement contexts is still poorly understood, but it has been attributed to a number of factors, from immigrant selectivity effects to cultural protective factors.^10^ Research rarely distinguishes between different types of migrants, although it is an important distinction in relation to risk of mental disorders. Distinct from immigrants, refugees are generally considered involuntary migrants (ie, forced to migrate given fears of violence or persecution). Importantly, refugee children and youth are more likely to have endured traumatic experiences associated with mental health vulnerability.^11,12^

Despite these hypothesized differences, there is limited research on population-level patterns of mental disorder prevalence for first- and second-generation immigrant and refugee children and youth and how such patterns may vary by age and sex and across mental disorders. The objective of the current study was to estimate the administrative data–derived diagnostic prevalence of mental disorders (conduct, attention-deficit/hyperactivity disorder [ADHD], and mood/anxiety) for first- and second-generation immigrant and refugee as well as nonimmigrant children and youth in British Columbia, Canada. Leveraging population-level administrative data, diagnostic prevalence estimates were calculated across 3 age groups (3-5 years, 6-12 years, and 13-19 years) and stratified by generation status (first-generation, second-generation, or nonimmigrant), migration category (immigrant or refugee for first- and second-generation children) and sex (female and male), all while accounting for years in British Columbia.

## Methods

### Data Sources and Study Population

The study was conducted using population-level, linked, longitudinal data spanning 2 decades in British Columbia. The study population (N = 470 464), identified using data from the British Columbia Ministry of Education^13^ and the Ministry of Health,^14,15^ captured all children and youth, ages 0 to 19 years, registered in the 10 largest school districts in the province at some point between 1996 and 2016. These 10 districts were selected because they captured the vast majority of the immigrant population in British Columbia.^16^ Children were included in the study cohort at birth or later and were dropped from the cohort at age 20 years. Only children registered with British Columbia’s universal health insurance program (Medical Services Plan [MSP]) for at least 275 days in at least 1 year between 1996 and 2016 were included, a common criterion to indicate residence in British Columbia.^17,18,19^ This criterion was applied again to age group–specific analyses.

Data from the British Columbia Ministry of Health^20,21^ (practitioner billing records, hospitalizations); BC PharmaNet^15^ (prescription drug dispensations); and Immigration, Refugees, and Citizenship Canada’s (IRCC) Permanent Resident Database^22^ (migration records) were requested and linked via Population Data BC^23^ using a probabilistic-deterministic approach (linkage rate, 98.4%). The study was approved by the University of British Columbia Behavioural Research Ethics Board (H10-01154). Informed consent was not required for this study given that data were administrative and deidentified. This study followed the Strengthening the Reporting of Observational Studies in Epidemiology (STROBE) reporting guideline.

### Study Variables

#### Mental Disorders

We adapted criteria created by the Manitoba Centre for Health Policy,^24^ which use a combination of *International Classification of Diseases, Ninth Revision, Clinical Modification* (*ICD-9-CM*) and *International Classification of Diseases, Tenth Revision *(*ICD-10*) codes from hospital discharge records and practitioner billing records to identify indicators of conduct disorder, ADHD, and mood/anxiety disorders. Some definitions also included drug dispensation information (eTables 1-3 in the Supplement). In line with previous research,^24^ we use the term *diagnostic prevalence* to signify that we are capturing prevalence through diagnoses documented in health service records. Some children and youth with a disorder may not access mental health services through the health care system or at all, and they would not be classified as having a disorder in the current study.

Dichotomous variables (1 and 0) indicated whether a child met criteria for each mental disorder at least once for each age range group (ages 3-5, 6-12, and 13-19 years). As the accuracy of MSP billings for very young children is low due to difficulty in assessment,^25,26^ diagnoses were not included prior to age 3 years.

A dichotomous comorbidity variable identified children diagnosed with 1 mental disorder (0) and children diagnosed with more than 1 of the 3 diagnoses (conduct, ADHD, mood/anxiety) examined in the current study (1). One common British Columbia–specific diagnostic code combines mood and anxiety disorders, making it impossible to distinguish these diagnoses. These disorders also often co-occur, and so as in other studies,^24^ the decision was made to combine mood and anxiety disorders.

#### Migration Variables

Children’s and their parents’ immigration status was identified through IRCC’s Permanent Resident Database. Children with their own IRCC records were coded as first-generation (ie, born outside of Canada), and those with at least 1 parent with an IRCC record were coded as second-generation (children were linked to their parents using MSP registration information). All others were coded as nonimmigrants. Children (or their parents) who arrived under the economic or family immigration categories were coded as immigrants. Children (or their parents) who arrived under the refugee category were coded as refugees. If parents had different immigrant and refugee categorizations, the refugee category was used given the potential salience of the refugee experience in children’s development.

#### Demographic Variables

Child’s biological sex at birth (or at immigration) and years in British Columbia variables came from MSP registration information. No data on gender were available. A variable that summed the number of years that a child was in British Columbia within an age range (eg, 6 to 12 years) was created to adjust for time spent in British Columbia.

### Statistical Analysis

Descriptive analyses showing sociodemographic characteristics for the study population were run overall and for each age group. To estimate the prevalence of conduct, ADHD, and mood/anxiety for each of the 3 age ranges, a series of log-binomial regression models were run in Stata version 16.1 (StataCorp) to identify adjusted coefficient values (using the binreg and lincom commands). These coefficient values were then exponentiated and multiplied by 100 to obtain the final percentage estimates (ie, prevalence values). Overall estimates were adjusted for years in British Columbia. Sex-stratified prevalence estimates were adjusted for years in British Columbia. Immigration status estimates included adjustments for sex and years in British Columbia.

Finally, the sample was restricted to individuals with a mental disorder diagnosis (conduct, ADHD, or mood/anxiety), and a series of log-binomial regression models were run (using Stata binreg and margin commands) to estimate the proportion of those with at least 1 comorbidity in each age range, stratified by sex and immigration status and adjusted for years in British Columbia.

We used 95% CIs to determine statistical significance. Analyses were undertaken from August 2020 to November 2021.

## Results

### Descriptive Statistics

A total of 470 464 children and youth in British Columbia were included in the study (227 217 [48.3%] female). Nonimmigrant children and youth represented 65.5% of the total study population (307 902 individuals). The other 34.55% of children and youth (161 697 individuals) migrated to Canada. Among those who migrated, 142 011 (87.8%) were first- or second-generation immigrants, and 19 686 (12.2%) were first- or second-generation refugees. Table 1 presents a demographic breakdown for each age range as well as the full study population.

**Table 1.** Demographic Characteristics of the Study Population

Characteristic	Study population, No. (%)^a^			
	Total (N = 470 464)	Age 3-5 y (n = 335 926)	Age 6-12 y (n = 427 476)	Age 13-19 y (n = 382 282)
Sex^b^				
Female	227 217 (48.30)	162 645 (48.42)	206 668 (48.35)	184 882 (48.36)
Male	243 243 (51.70)	173 279 (51.58)	220 805 (51.65)	197 397 (51.64)
Generation status				
First-generation	86 443 (18.37)	20 131 (5.99)	63 054 (14.75)	75 886 (19.85)
Second-generation	76 119 (16.18)	66 968 (19.94)	73 939 (17.30)	55 372 (14.49)
Nonimmigrant	307 902 (65.45)	248 827 (74.07)	290 483 (67.95)	251 024 (65.66)
Migration category^c^				
Immigrant	142 011 (87.83)	74 924 (86.75)	119 020 (87.41)	114 987 (88.10)
Refugee	19 686 (12.17)	11 444 (13.25)	17 139 (12.59)	15 539 (11.90)

^a^ Individuals were included in the study population for each age range if they were present in British Columbia for at least 1 of the years within the age range (see Methods section for further details).

^b^ A few individuals had missing data for sex.

^c^ Identifies migration category for first- and second-generation children. Note that less than 1% arrived under other categorizations and were treated as missing.

### Diagnostic Prevalence Estimates

Table 2 presents diagnostic prevalence estimates adjusted for years in British Columbia. Any group differences presented here assume nonoverlapping confidence intervals unless otherwise specified. Overall, conduct and ADHD estimates were highest in children aged 6 to 12 years. Diagnostic prevalence estimates were highest for mood/anxiety disorders in children aged 13 to 19 years. The diagnostic prevalence of both conduct disorder and ADHD was higher for male participants across each age range. Estimates of mood/anxiety were also higher for male participants aged 3 to 5 years and 6 to 12 years, but this changed for those aged 13 to 19 years, in which estimates were markedly higher for female participants.

**Table 2.** Diagnostic Prevalence Estimates of Conduct, ADHD, and Mood/Anxiety Stratified by Age Range, Sex, and Immigration Status, Adjusted for Years in British Columbia

Subgroup	Prevalence estimate (95% CI), %^a^								
	Conduct			ADHD			Mood/anxiety		
	Age 3-5 y	Age 6-12 y	Age 13-19 y	Age 3-5 y	Age 6-12 y	Age 13-19 y	Age 3-5 y	Age 6-12 y	Age 13-19 y
**Total population**									
Overall	2.88 (2.81-2.94)	6.22 (6.13-6.30)	3.25 (3.17-3.32)	1.16 (1.11-1.22)	8.22 (8.13-8.32)	5.69 (5.60-5.79)	1.73 (1.68-1.78)	7.82 (7.72-7.91)	21.12 (21.00-21.31)
Female	1.99 (1.92-2.06)	3.64 (3.55-3.73)	2.24 (2.16-2.32)	0.54 (0.51-0.58)	4.23 (4.14-4.33)	3.38 (3.29-3.48)	1.57 (1.51-1.64)	6.86 (6.74-6.99)	25.54 (25.28-25.79)
Male	3.71 (3.61-3.80)	8.64 (8.50-8.77)	4.19 (4.08-4.30)	1.74 (1.66-1.83)	11.98 (11.82-12.14)	7.86 (7.71-8.01)	1.88 (1.81-1.95)	8.71 (8.58-8.85)	16.97 (16.76-17.17)
**Immigrant**									
First generation									
Overall	1.53 (1.33-1.76)	2.72 (2.56-2.90)	1.16 (1.07-1.26)	0.82 (0.70-0.97)	4.30 (4.10-4.51)	2.13 (2.01-2.25)	1.62 (1.41-1.86)	4.78 (4.55-5.01)	11.07 (10.80-11.36)
Female	1.06 (0.92 (1.22)	1.37 (1.21-1.55)	0.80 (0.73-0.87)	0.39 (0.33-0.46)	1.73 (1.56-1.92)	1.07 (0.95-1.19)	1.47 (1.28-1.69)	4.16 (3.88-4.47)	12.83 (12.39-13.28)
Male	1.98 (1.72-2.28)	3.98 (3.72-4.27)	1.50 (1.38-1.63)	1.24 (1.05-1.45)	6.69 (6.35-7.04)	3.10 (2.91-3.31)	1.76 (1.53-2.02)	5.33 (5.02-5.68)	9.45 (9.10-9.83)
Second generation									
Overall	2.07 (1.96-2.20)	4.29 (4.13-4.46)	2.23 (2.08-2.39)	0.89 (0.81-0.97)	5.94 (5.75-6.14)	3.72 (3.53-3.91)	1.78 (1.67-1.89)	5.99 (5.80-6.19)	15.99 (15.60-16.40)
Female	1.42 (1.33-1.52)	2.54 (2.41-2.69)	1.52 (1.41-1.64)	0.41 (0.37-0.46)	2.73 (2.54-2.93)	1.93 (1.74-2.14)	1.61 (1.50-1.73)	5.29 (5.02-5.56)	18.86 (18.25-19.50)
Male	2.66 (2.50-2.82)	5.95 (5.67-6.23)	2.86 (2.66-3.07)	1.32 (1.21-1.45)	8.81 (8.49-9.14)	5.30 (5.00-5.61)	1.93 (1.80-2.06)	6.62 (6.34-6.91)	13.45 (12.96-13.97)
**Refugee**									
First generation									
Overall	2.34 (1.68-3.27)	4.37 (3.80-5.02)	1.78 (1.46-2.16)	0.58 (0.33-1.02)	4.05 (3.54-4.65)	2.44 (2.09-2.84)	2.35 (1.69-3.26)	6.34 (5.64-7.14)	16.12 (15.15-17.15)
Female	1.61 (1.15-2.25)	2.46 (2.07-2.91)	1.22 (1.00-1.48)	0.27 (0.15-0.49)	2.28 (1.96-2.66)	1.54 (1.29-1.85)	2.13 (1.53-2.97)	4.46 (3.75-5.31)	19.09 (17.27-21.12)
Male	3.01 (2.16-4.20)	5.75 (4.87-6.79)	2.30 (1.89-2.79)	0.86 (0.49-1.51)	6.21 (5.33-7.22)	3.49 (2.92-4.17)	2.55 (1.83-3.54)	5.71 (4.81-6.78)	12.54 (11.36-13.85)
Second generation									
Overall	2.26 (1.97-2.60)	4.22 (3.83-4.64)	1.72 (1.42-2.09)	1.05 (0.88-1.27)	6.20 (5.63-6.57)	3.67 (3.25-4.14)	2.58 (2.27-2.94)	6.49 (6.01-7.02)	16.91 (15.96-17.92)
Female	1.56 (1.35-1.80)	2.06 (1.69-2.51)	1.19 (0.98-1.44)	0.49 (0.41-0.60)	2.76 (2.34-3.27)	2.36 (2.04-2.72)	2.34 (2.05-2.68)	5.44 (4.82-6.15)	21.01 (19.17-23.04)
Male	2.92 (2.53-3.35)	6.25 (5.61-6.97)	2.23 (1.85-2.71)	1.58 (1.31-1.90)	9.22 (8.45-10.05)	5.33 (4.64-6.13)	2.80 (2.46-3.19)	7.48 (6.77-8.27)	13.81 (12.61-15.11)
**Nonimmigrant**									
Overall	3.14 (3.07-3.23)	7.03 (6.93-7.13)	4.00 (3.89-4.08)	1.27 (1.21-1.34)	9.20 (9.08-9.31)	7.04 (6.91-7.16)	1.69 (1.64-1.74)	8.53 (8.42-8.65)	24.54 (24.34-24.76)
Female	2.18 (2.10-2.26)	4.17 (4.06-4.28)	2.74 (2.64-2.85)	0.60 (0.55-0.64)	4.89 (4.77-5.01)	4.27 (4.15-4.40)	1.54 (1.47-1.61)	7.46 (7.32-7.62)	29.76 (29.43-30.06)
Male	4.07 (3.96-4.19)	9.75 (9.59-9.92)	5.17(5.03-5.32)	1.91 (1.82-2.02)	13.29 (13.11-13.48)	9.66 (9.47-9.86)	1.84 (1.76-1.91)	9.56 (9.39-9.72)	19.53 (19.28-19.81)

Abbreviation: ADHD, attention-deficit/hyperactivity disorder.

^a^ All prevalence estimates in the table are adjusted by number of years of residence in British Columbia across the full age range. Estimates are based on individual-level data (ie, unique individuals). Overall prevalence estimates (conduct, ADHD, or mood/anxiety), adjusted for years in British Columbia, for the 3-to-5–year, 6-to-12–year, and 13-to-19–year age ranges were 5.29%, 16.07%, and 24.34%, respectively.

First-generation immigrant children and youth generally showed the lowest diagnostic prevalence estimates for conduct disorder, ADHD, and mood/anxiety disorder, while nonimmigrant children and youth generally showed the highest estimates (conduct disorder: eg, age 6-12 years, first-generation immigrant, 2.72% [95% CI, 2.56%-2.90%] vs nonimmigrant, 7.03% [95% CI, 6.93%-7.13%]; ADHD: eg, age 6-12 years, first-generation immigrant, 4.30% [95% CI, 4.10%-4.51%] vs nonimmigrant, 9.20% [95% CI, 9.08%-9.31%]; mood/anxiety disorders: eg, age 13-19 years, first-generation immigrant, 11.07% [95% CI, 10.80%-11.36%] vs nonimmigrant, 24.54% [95% CI, 24.34%-24.76%]). The main exception to this was in children aged 3 to 5 years for mood/anxiety disorder and ADHD, in which diagnostic prevalence estimates were small and showed less variation. First- and second-generation refugees had diagnostic prevalence estimates that were generally similar, with overlapping 95% CIs with a few exceptions: first-generation refugees aged 6 to 12 years and 13 to 19 years had lower diagnostic prevalence estimates for ADHD (eg, age 6-12 years: 4.05% [95% CI, 3.54%-4.65%]) than second-generation refugees (age 6-12 years: 6.20% [95% CI, 5.63%-6.57%]) and immigrants (age 6-12 years: 5.94% [95% CI, 5.75%-6.14%]). First-generation refugees had higher estimates of mood/anxiety disorders in the 13-to-19–years age range (16.12% [95% CI, 15.15%-17.15%]) relative to first-generation immigrants (11.07% [95% CI, 10.80%-11.36%]). Second-generation refugee children had the highest diagnostic prevalence estimates for mood/anxiety disorder in the 3-to-5–year age range relative to the first- and second-generation immigrant and non-immigrant groups (eg, second-generation refugee, 2.58% [95% CI, 2.27%-2.94%]; second-generation immigrant, 1.78% [95% CI, 1.67%-1.89%]) (Figure).

**Figure.**
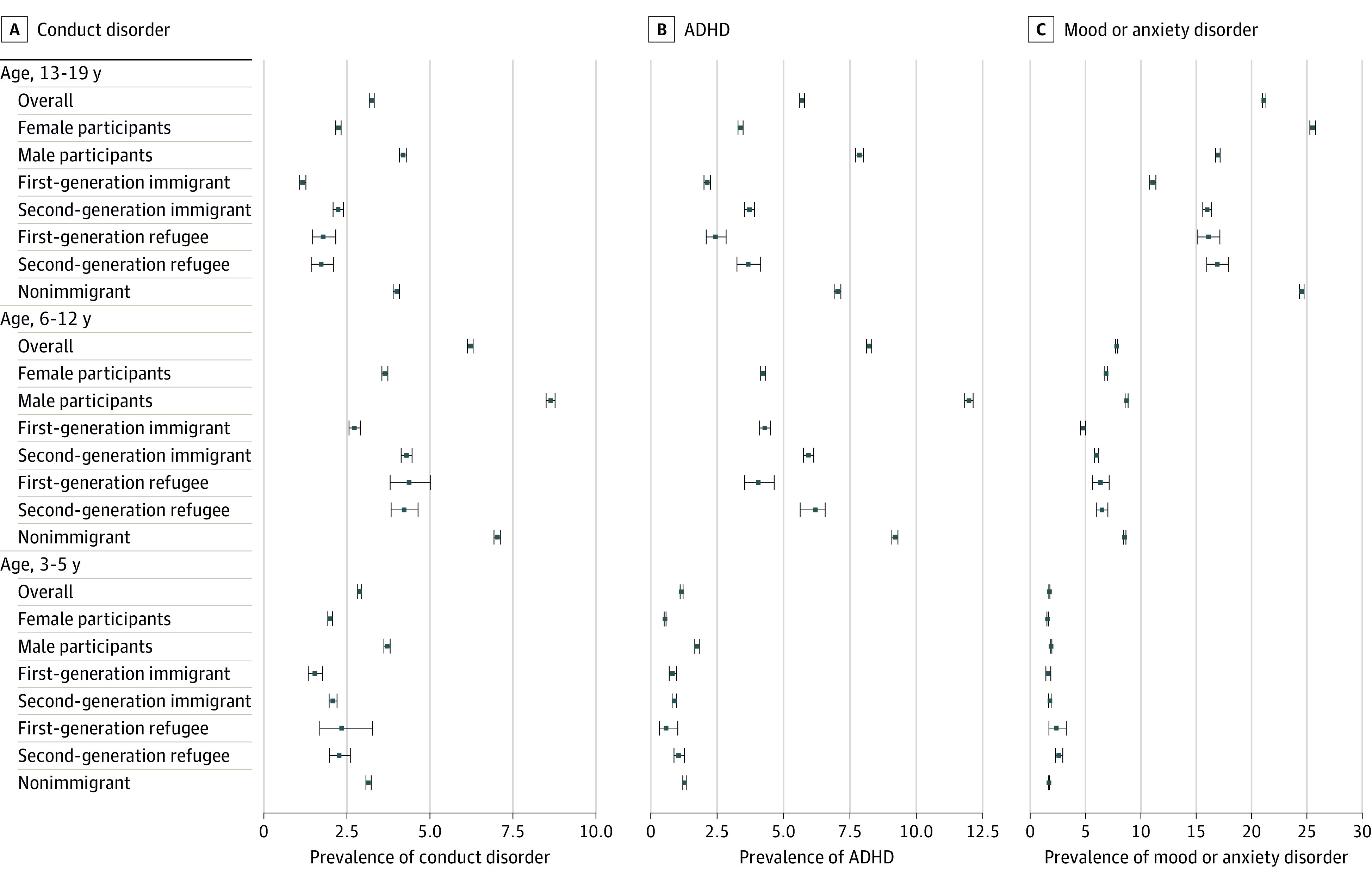
Diagnostic Prevalence Estimates of Conduct, Attention-Deficit/Hyperactivity Disorder (ADHD), Mood/Anxiety Disorders Stratified by Age Range, Sex, and Immigration Status, Adjusted for Years in British Columbia Estimates are based on individual level data (ie, unique individuals).

### Comorbidity Estimates

Table 3 presents the estimated percentages of comorbidities, stratified by age range, sex, and immigration status (adjusted for years in British Columbia). Male participants had a higher percentage of comorbidities across age ranges than female participants. A smaller proportion of first- and second-generation immigrant and refugee children had comorbidities vs nonimmigrant children, although there were some overlapping 95% CIs among children aged 3 to 5 years. Refugee children had a similar or lower proportion of comorbidities relative to immigrant children.

**Table 3.** Estimated Percentage of Children Diagnosed With Conduct, ADHD, and Mood/Anxiety With at Least 1 Comorbidity, Stratified by Age Range, Sex, and Immigration Status, Adjusted for Years in British Columbia

Group	Estimated proportion with ≥1 comorbidity, % (95% CI)^a^		
	Age 3-5 y (n = 16 129)	Age 6-12 y (n = 59 958)	Age 13-19 y (n = 71 872)
**Total population**			
Overall	13.62 (13.04-14.19)	31.27 (30.85-31.69)	19.26 (18.90-19.63)
Female	9.71 (8.93-10.49)	23.62 (23.02-24.23)	13.40 (13.02-13.78)
Male	15.74 (14.99-16.48)	35.27 (34.74-35.80)	25.76 (25.20-26.32)
**Immigrant**			
First generation			
Overall	11.35 (8.64-14.07)	21.87 (20.51-23.23)	12.30 (11.48-13.11)
Female	8.11 (6.10-10.12)	16.30 (15.23-17.37)	8.42 (7.85- 9.00)
Male	13.16 (10.01-16.31)	24.45 (22.93-25.97)	16.26 (15.18-17.33)
Second generation			
Overall	11.32 (10.05-12.59)	24.02 (23.03-25.01)	14.78 (13.91-15.65)
Female	8.03 (6.98-9.09)	17.99 (17.15-18.82)	10.09 (9.47-10.72)
Male	13.03 (11.55-14.51)	26.97 (25.85-28.09)	19.48 (18.33-20.62)
**Refugee**			
First generation			
Overall	7.47 (1.73-13.21)	18.12 (14.92-21.33)	9.36 (7.62-11.10)
Female	5.32 (1.23-9.42)	13.98 (11.50-16.45)	6.59 (5.37-7.81)
Male	8.64 (2.02-15.25)	20.96 (17.27-24.65)	12.72 (10.37-15.07)
Second generation			
Overall	11.06 (8.30-13.82)	23.39 (21.10-25.68)	12.83 (10.96-14.71)
Female	7.95 (5.90-10.00)	17.47 (15.73-19.20)	8.94 (7.64-10.25)
Male	12.90 (9.67-16.12)	26.20 (23.65-28.75)	17.26 (14.76-19.77)
**Nonimmigrant**			
Overall	14.20 (13.55-14.85)	32.81 (32.34-33.28)	20.49 (20.08-20.90)
Female	10.13 (9.29-10.96)	24.79 (24.15-25.44)	14.29 (13.88-14.71)
Male	16.43 (15.60-17.26)	37.19 (36.60-37.77)	27.59 (26.97-28.20)

Abbreviation: ADHD, attention-deficit/hyperactivity disorder.

^a^ Restricted to children with at least 1 mental disorder diagnosis (conduct, ADHD, mood/anxiety) within the age range. All estimates in the table are adjusted to represent presence in British Columbia for the full age range (ie, from 3-5 years, 6-12 years, and 13-19 years, respectively). Comorbidities were restricted to those diagnoses captured in the current study (ie, conduct disorder, ADHD, mood/anxiety disorder).

## Discussion

We found a general pattern of lower diagnostic prevalence estimates for first- and second-generation immigrant and refugee children and youth vs their nonimmigrant counterparts. This was consistent with findings in Ontario, which found lower mental disorder prevalence for immigrant children and youth than for nonimmigrant children and youth.^9^ There are a number of systemic reasons why diagnostic mental disorder prevalence might be lower for first- and second-generation immigrant and refugee children. It may reflect differences in health service use and access, such as differences in treatment seeking and barriers to accessing services (eg, language skills). Health services are thought to be underutilized by immigrant groups in Canada, especially those originating from East Asia.^27^ A previous study in Ontario found mental health–related service contact for those with a mental disorder to be lower for immigrant vs nonimmigrant populations.^9^

Cultural differences may also play an important role: theory, research, and practices associated with mental health are largely grounded in Western conceptualizations of mental disorders and symptomatology, which may not accurately represent the needs of those from differing cultural backgrounds.^28,29^ First- and second-generation immigrant and refugee children and youth may also have protective factors that could play a role in explaining prevalence differences. These may include strong ethnic identity and cultural support systems^30,31^ and the notion that some immigrants may be particularly well-resourced and healthy due to sociodemographic factors associated with immigrant selectivity.^32,33^ The current study offers insight into differences in the diagnostic mental disorder prevalence of children and youth in British Columbia, although more work is needed to understand cultural and systemic patterns underlying these differences, which remain a critical area for further research.

### Differences Among Immigrant and Refugee Children and Youth

We identified notable differences in mental disorder prevalence based on generation status, with first-generation children overall having the lowest diagnostic prevalence estimates (vs second-generation children). These findings suggest that immigrant children and youth who (themselves or their families) have been in Canada for longer seem to have an increased likelihood of being diagnosed with a mental disorder (a likelihood more in line with the nonimmigrant population). This pattern has been observed across a number of child and youth developmental outcomes^34^ and raises questions about why we see patterns of worsening mental health over time spent in the country.^35^ One possibility is that this trend is representative of greater comfort and/or ability in accessing services over time, an area that warrants further investigation.

Some differences in prevalence for immigrant and refugee groups were also observed. First-generation refugee youth had higher estimates of mood/anxiety disorder in the 13-to-19–year age range relative to the first-generation immigrant group. Differences in prevalence estimates across immigrant and refugee children and youth are important to note given that they may have had very different premigration and postmigration experiences. Refugee youth are more likely to have experienced trauma, which may play a strong and lasting role in the psychological outcomes of refugee children.^11,12^ This underscores the need to consider the potential for different needs across these 2 groups in mental health service planning.

Second-generation refugee children had the highest prevalence estimates for mood/anxiety disorders in the 3-to-5–year age range relative to first- and second-generation immigrant children (confidence intervals overlapped for first- and second-generation refugees). Trauma is often associated with refugee experiences, but this finding aligns with previous research indicating that trauma can have an intergenerational impact and may play a role in the psychological outcomes of children of refugees (ie, second-generation refugee children).^36^ Mood/anxiety diagnoses in this age range are generally very low but important to consider, as research has linked poor early emotional functioning in refugee children with worsening developmental outcomes over time.^37^

Female individuals had higher diagnostic prevalence of mood/anxiety disorder in the 13-to-19–year age range; however, male individuals had higher prevalence in the 6-to-12–year age range. This male-female reversal in prevalence is consistent with some other research: Spady and colleagues^38^ found a similar male-female reversal from childhood to adolescence in the prevalence of depression and anxiety. Studies using diagnostic interviews have found a male-female reversal for depression.^9,39^ The marked increase in depression and anxiety in female children and youth has been partly attributed to pubertal transitions,^38,40^ but the question remains as to why males would show higher levels of mood/anxiety disorder in childhood. One possibility is that male children have increased contact with mental health practitioners in childhood because of their higher prevalence of externalizing disorders at that stage and may therefore be more likely to receive additional mental disorder diagnoses. It warrants further investigation, but this hypothesis is corroborated by the higher levels of comorbidity in male participants found in the current study.

### Comorbidities

A substantial proportion of children and youth with a diagnosis of ADHD, conduct disorder, or mood/anxiety disorders were found to have at least 1 other diagnosis (ie, of ADHD, conduct disorder, or mood/anxiety disorder): nearly one-third of children aged 6 to 12 years and one-fifth of youth aged 13 to 19 years. In Manitoba, Spady and colleagues^38^ found that 20.8% of children (aged 0 to 17 years) with a psychiatric disorder were identified as having more than 1 in the same year.^38^ The current study builds on this work by identifying that comorbidity varied by migration status such that first- and second-generation immigrant and refugee children had fewer comorbidities than nonimmigrant children. The potential mechanisms underlying these differences are unclear but provide an important area for further investigation.

We also found that male children and youth were more likely than female children and youth to have comorbidity. Although consistent with the findings of Spady and colleagues,^38^ we are careful to note that the sex differences in comorbidity may be partly due to 2 factors. First, mood and anxiety disorders were combined in the current study because they could not be differentiated in all cases in the data. It is possible that female children and youth had fewer comorbidities because mood and anxiety disorders were already combined. Second, male children and youth may have had higher proportions of comorbidities simply because they are more likely to have ADHD and conduct diagnoses.

### Limitations

This study has limitations. Notably, the prevalence estimates in the study captured diagnostic prevalence based on health service utilization. True prevalence estimates would capture the actual number of children in British Columbia with mental disorders, including those who have not yet accessed mental health services or who accessed services not billed through the province’s health insurance program. Additionally, there is also the possibility of overdiagnosis (ie, false-positive diagnoses) recorded in health service utilization records, representing the potential for bias on the other end of the spectrum. Although the data were not available in the current study to make any determination of bias, there is evidence from other work to suggest that mental health service contact can differ by mental health disorder class and immigration background.^9^ As others have highlighted, there is a distinct need for research that captures the broader prevalence picture to support informed policy decisions.^41^

There are cultural limitations to note. First, we were unable to account for ethnicity or country of origin. Therefore, we could not examine ethnic and cultural differences across immigrant, refugee, and nonimmigrant groups. Second, although the international classification system (eg, *ICD*) offers clear strengths in terms of comparability across contexts, researchers have raised concerns regarding the validity of an international coding system across cultural contexts.^42^ More work is needed to validate and contextualize our understanding of *ICD* codes across cultural contexts.

## Conclusions

In this study, immigrant and refugee children generally had lower diagnostic prevalence rates of conduct disorder, ADHD, and mood/anxiety disorders, although there were important differences by sex, age, and first- vs second-generation status. The study makes an important contribution to our understanding of mental disorder prevalence for first- and second-generation immigrant and refugee children and youth. To our knowledge, this is the first study to provide population-level mental disorder prevalence estimates that compare immigrant, refugee, and nonimmigrant groups in British Columbia. It also enhances our understanding of mental disorder prevalence for refugee children and youth more broadly, a group that is rarely captured and often underrepresented in general population surveys. This information is particularly important for health service planning and to inform policy. Future research is needed to tease apart the nuances underlying these differences, including the examination of cultural and systemic differences, such as cultural differences in symptomatology, presentation, and etiologies as well as systemic barriers to accessing health services. Future work should also consider mental health protective factors for certain immigrant and refugee children/youth (eg, ethnic identity and cultural support systems). Understanding differences in mental disorder prevalence estimates for subpopulations is a critical first step in ensuring that all children’s mental health needs are met.

## Notes

Supplement. **eTable 1.** Mental Health Definitions **eTable 2.** List of Chemical/Generic Drug Names for ADHD **eTable 3.** List of Chemical/Generic Drug Names for Mood/Anxiety Disorders (Depression, Anxiety, or Bipolar Disorder)
