# Supplementary material for: Prevalence of Mental Health Disorders Among Immigrant, Refugee, and Nonimmigrant Children and Youth in British Columbia, Canada
Source: JAMA Netw Open. 2022 Feb 15;5(2):e2144934. doi: 10.1001/jamanetworkopen.2021.44934 (PMC8848209; doi:10.1001/jamanetworkopen.2021.44934)
Supplement: Supplement. — eTable 1. Mental Health Definitions eTable 2. List of Chemical/Generic Drug Names for ADHD eTable 3. List of Chemical/Generic Drug Names for Mood/Anxiety Disorders (Depression, Anxiety, or Bipolar Disorder) [file jamanetwopen-e2144934-s001.pdf]

Supplemental Online Content

Gadermann AM, Gagné Petteni M, Janus M, Puyat JH, Guhn M, Georgiades K. Prevalence of mental health disorders among immigrant, refugee, and nonimmigrant children and youth in British Columbia, Canada. *JAMA Netw Open*. 2022;5(2):e2144934. doi:10.1001/jamanetworkopen.2021.44934

eTable 1. Mental Health Definitions

eTable 2. List of Chemical/Generic Drug Names for ADHD

eTable 3. List of Chemical/Generic Drug Names for Mood/Anxiety Disorders (Depression, Anxiety, or Bipolar Disorder)

This supplemental material has been provided by the authors to give readers additional information about their work.

© 2022 Gadermann AM et al. *JAMA Network Open.*

eTable 1. Mental Health Definitions

<table>
<tr><td colspan="2">Attention-Deficit Hyperactivity Disorder (ADHD)
ADHD is a neurobehavioural developmental disorder that is characterized by a persistent pattern of impulsiveness, hyperactivity and absence of attention in children (Chartier et al., 2016).</td></tr>
<tr><td>Changes made to the original MCHP ADHD definition
• Replaced MCHP drug list with a drug list development by pharmacists in BC.
• Removed criterion that was based solely on drug dispensation
• Replaced three-year lookback window in criterion three to a same year criterion to prevent biased estimates (due to group differences in presence in BC for the lookback period).</td><td>ADHD technical definition
1. 1+ hospitalizations with diagnosis of hyperkinetic syndrome (ICD-9-CM code 314 or ICD-10 code F90) in one calendar year,
2. OR, 1+ physician claims with diagnosis of hyperkinetic syndrome (ICD-9-CM code 314) in one calendar year,
3. OR, 1 Rx for ADHD drugs (*see chemical/generic drug name list below) in one calendar year with diagnosis of hyperkinetic syndrome (ICD-9-CM code 314 or ICD-10 code F90) in the same year.</td></tr>
<tr><td colspan="2">Conduct disorder

Conduct disorder is characterized by a repetitive and persistent pattern of dissocial, aggressive or defiant behaviour, which is enduring and more severe than ordinary childish mischief or adolescent rebelliousness (Chartier et al., 2016).</td></tr>
<tr><td>Changes made to the original MCHP Conduct definition
• No changes made</td><td>Conduct technical definition
1. 1+ hospitalizations with diagnosis of conduct disorders (ICD-9: 312 or ICD-10: All F91 codes except F91.3 - oppositional disorder)
2. OR, 1+ physician visits with a diagnosis of conduct disorders (ICD-9: 312)</td></tr>
<tr><td colspan="2">Mood/anxiety disorders

Mood/anxiety disorders consist of a broad group of mental disorders including depressive (depressed mood and lack of interest in activities), bipolar (elevated mood and increased energy), and anxiety disorders (excessive fear, anxiety or worry and often avoidance; Chartier et al., 2016).</td></tr>
<tr><td>Changes made to the original MCHP Mood/anxiety definition
• Replaced MCHP drug list with a drug list development by pharmacists in BC.
• Added a BC-specific MSP-only diagnostic code 50B (depression/anxiety) to criterion 3.
• Included ICD codes associated with childhood specific emotional disorders (F93 and 313)</td><td>Mood/anxiety technical definition
1. 1+ hospitalizations with one or more of the following diagnosis codes: ICD9 296 (Bipolar; manic depressive); 300.0, 300.2, 300.3, 300.4, 300.7 (anxiety disorders), 309 (adjustment reaction; depression/anxiety); 313 (disturbance of emotions specific to childhood and adolescence); or 311 (depression disorder); ICD10 F31 (bipolar affective disorder), F32 (Depressive episode), F33 (Recurrent depressive disorder), F341 (dysthmymic; mood), F38.0, F38.1 (other mood), F40 (Phobic anxiety disorders), F41.0 (panic disorder), F41.1 (Gen. anxiety disorder), F41.2 (mixed anxiety, depressive), F41.3 (mixed anxiety, depressive), F41.8 (other anxiety), F41.9 (anxiety), F42 (OCD), F43.1 (PTSD), F43.2 (adjustment; depression/anxiety), F43.8 (severe stress reaction), F53.0 (postpartum other mental/behavioral), or F93 (emotional disorders with onset specific to childhood)
2. OR, 1+ hospitalizations with one or more of the following diagnosis codes: ICD9 300 (anxiety disorders); ICD10 F32 (depressive episode), F341 (dysthymic; mood), F40 (Phobic anxiety disorder), F41 (other anxiety), F42 (OCD), F44 (dissociative disorders), F45.0 (somatization disorder), F45.1 (undifferentiated somatoform), F45.2 (hypochondriachal), F48</td></tr>
</table>

© 2022 Gadermann AM et al. *JAMA Network Open.*

	(other neurotic disorders), F68.0 (elaboration of physical symptoms) or F99 (unspecified mental disorder) AND one or more Rx for a mood/anxiety disorder (i.e., anxiety, depression, or bipolar disorders; see list of chemical/generic drug names listed below).<br>3. OR, 1+ physician visits with one or more of the following diagnosis codes: ICD9 296 (bipolar, manic depression), 311 (depressive disorder), 50B (depression/anxiety)<br>4. OR, 1+ physician visits with a diagnosis code: ICD9 300 (anxiety disorders) AND one or more Rx for a mood/anxiety disorder (i.e., anxiety, depression, or bipolar disorders; see list of chemical/generic drug names listed below).<br>5. OR, 3+ physician visits with a diagnosis code: ICD9 300 (anxiety disorder); 309 (adjustment reaction; anxiety/depression).
**Note.** Definition discrepancies in the use of ICD-9 and ICD-10 diagnostic coding are due to differences in data availability. Currently in the province of BC, Canada, outpatient/physician visit claim data are in ICD-9 format only. Hospitalization data captures both ICD-9 and ICD-10 formats, depending on the data year.	

© 2022 Gadermann AM et al. *JAMA Network Open.*

eTable 2. List of Chemical/Generic Drug Names for ADHD

**Note: The two columns below represent variations in spelling/names. Either variation is acceptable in our definition.**		
AMFETAMINE	OR	Amphetamine
ATOMOXETINE	OR	ATOMOXETINE HCL
BUPROPION	OR	BUPROPION HCL
CLONIDINE	OR	CLONIDINE HCL
CLONIDINE AND DIURETICS	OR	
DESIPRAMINE	OR	DESIPRAMINE HCL
DEXAMFETAMINE	OR	
GUANFACINE	OR	GUANFACINE HCL
IMIPRAMINE	OR	IMIPRAMINE HCL
LISDEXAMFETAMINE	OR	LISDEXAMFETAMINE DIMESYLATE
METHYLPHENIDATE	OR	METHYLPHENIDATE HCL
MODAFINIL	OR	
NORTRIPTYLINE	OR	NORTRIPTYLINE HCL
RISPERIDONE	OR	RISPERIDONE MICROSPHERES
VENLAFAXINE	OR	VENLAFAXINE HCL

© 2022 Gadermann AM et al. *JAMA Network Open.*

eTable 3. List of Chemical/Generic Drug Names for Mood/Anxiety Disorders (Depression, Anxiety, or Bipolar Disorder)

**Note: The two columns below represent variations in spelling/names. Either variation is acceptable in our definition.**		
ALPRAZOLAM	OR	
AMITRIPTYLINE	OR	AMITRIPTYLINE PAMOATE; AMITRIPTYLINE HCL; PERPHENAZINE / AMITRIPTYLINE HCL
ARIPIPRAZOLE	OR	
ASENAPINE	OR	ASENAPINE MALEATE
BENZTROPINE	OR	BENZTROPINE MESYLATE
BROMAZEPAM	OR	
BUPROPION HCL	OR	
CARBAMAZEPINE	OR	
CHLORDIAZEPOXIDE	OR	CHLORDIAZEPOXIDE/CLIDINIUM BR; CHLORDIAZEPOXIDE HCL
CHLORPROMAZINE	OR	CHLORPROMAZINE HCL
CLOBAZAM	OR	
CITALOPRAM HYDROBROMIDE	OR	
CLOMIPRAMINE	OR	CLOMIPRAMINE HCL
CLONAZEPAM	OR	
CLORAZEPATE	OR	CLORAZEPATE DIPOTASSIUM
CLOZAPINE	OR	
DESIPRAMINE	OR	DESIPRAMINE HCL
DESVENLAFAXINE SUCCINATE	OR	
DIAZEPAM	OR	DIAZEPAM (IN SOYBEAN OIL)
DIVALPROX	OR	DIVALPROEX SODIUM
DOMPERIDONE INJ 50MG	OR	DOMPERIDONE MALEATE
DOXEPIN	OR	DOXEPIN HCL
DROPERIDOL	OR	
DULOXETINE HCL	OR	
ESCITALOPRAM	OR	
ESCITALOPRAM OXALATE	OR	
FLUOXETINE	OR	FLUOXETINE HCL
FLUPENTHIXOL	OR	
FLUPHENAZINE	OR	FLUPHENAZINE HCL; FLUPHENAZINE DECANOATE
FLURAZEPAM	OR	FLURAZEPAM HCL
FLUVOXAMINE	OR	FLUVOXAMINE MALEATE
GABAPENTIN	OR	
HALOPERIDOL	OR	HALOPERIDOL DECANOATE; HALOPERIDOL LACTATE
IMIPRAMINE	OR	IMIPRAMINE HCL
LAMOTRIGINE	OR	
LITHIUM SALTS	OR	LITHIUM CITRATE; LITHIUM CARBONATE
LORAZEPAM	OR	
LOXAPINE	OR	LOXAPINE HCL; LOXAPINE SUCCINATE
LURASIDONE	OR	LURASIDONE HCL
METHOTRIMEPRAZINE	OR	METHOTRIMEPRAZINE HCL; METHOTRIMEPRAZINE MALEATE
MIDAZOLAM	OR	MIDAZOLAM HCL; MIDAZOLAM HCL/PF
MIRTAZAPINE	OR	
MOCLOBEMIDE	OR	
NITRAZEPAM	OR	
NORTRIPTYLINE	OR	NORTRIPTYLINE HCL
OLANZAPINE	OR	
OXAZEPAM	OR	
OXCARBAZEPINE	OR	
PALIPERIDONE	OR	PALIPERIDONE PALMITATE
PAROXETINE	OR	PAROXETINE HCL
PERPHENAZINE	OR	PERPHENAZINE / AMITRIPTYLINE HCL
PHENELZINE	OR	PHENELZINE SULFATE
PIMOZIDE	OR	
PREGABALIN	OR	

© 2022 Gadermann AM et al. *JAMA Network Open.*

PROCHLORPERAZINE	OR	PROCHLORPERAZINE MALEATE
QUETIAPINE FUMARATE	OR	
RISPERIDONE	OR	RISPERIDONE MICROSPHERES
SERTRALINE	OR	SERTRALINE HCL
TEMAZEPAM	OR	
THIORIDAZINE	OR	THIORIDAZINE HCL
THIOTHIXENE	OR	
TRANYLCYPROMINE	OR	TRANYLCYPROMINE SULFATE
TRAZODONE	OR	TRAZODONE HCL
TRIAZOLAM	OR	
TRIFLUOPERAZINE	OR	TRIFLUOPERAZINE HCL; ISOPRIPAMIDE/TRIFLUOP HCL
TRIMIPRAMINE	OR	TRIMIPRAMINE MALEATE
VALPROATE	OR	VALPROIC ACID (AS SODIUM SALT); VALPROIC ACID
VENLAFAXINE	OR	VENLAFAXINE HCL;
VORTIOXETINE	OR	VORTIOXETINE HYDROBROMIDE
ZIPRASIDONE	OR	ZIPRASIDONE HCL
ZUCLOPENTHIXOL	OR	ZUCLOPENTHIXOL HCL; ZUCLOPENTHIXOL ACETATE; ZUCLOPENTHIXOL DECANOATE

© 2022 Gadermann AM et al. *JAMA Network Open.*
